# CT–Pathology Size Discordance and Size-Threshold–Defined Potential Overtreatment in Early-Stage Lung Cancer: Restricted Cubic Spline Analysis, Decision Curve Analysis, and Bootstrap Validation in 1096 Patients

**DOI:** 10.3390/cancers18071118

**Published:** 2026-03-30

**Authors:** Hao Xu, Han Zhang, Shilin Li, Linyou Zhang

**Affiliations:** Department of Thoracic Surgery, The Second Affiliated Hospital of Harbin Medical University, #148 Baojian Road, Harbin 150001, China

**Keywords:** non-small cell lung cancer, computed tomography, sublobar resection, lobectomy, overtreatment, measurement bias, decision curve analysis

## Abstract

Background: Current guidelines recommend lobectomy for tumors > 20 mm on CT, yet systematic CT–pathology size discordance may contribute to size-threshold–driven surgical decisions. We hypothesized that CT-based tumor diameter differs from pathological size near the 20 mm surgical boundary, potentially leading a proportion of patients to undergo more extensive resection than pathology would indicate under a size-only rule. Methods: We retrospectively analyzed 1096 patients undergoing thoracoscopic surgery for clinical stage I non-small cell lung cancer at a single center (2020–2024). CT–pathology agreement was assessed via Bland–Altman analysis. Optimal CT cut-off was identified using restricted cubic spline (RCS) modeling, internally validated with bootstrap resampling (B = 2000), and evaluated by decision curve analysis (DCA). Results: CT showed size-dependent bias: overestimation in small tumors (T1a: +4.21 mm) transitioning to underestimation in larger lesions (≥T2: −7.49 mm). At the 20 mm threshold, 15.8% of patients (n = 173) underwent lobectomy despite pathological size ≤ 20 mm (potential overtreatment). RCS modeling and bootstrap-optimized DCA identified 23 mm as the candidate revised threshold. Adopting CT > 23 mm would reclassify 108 patients from lobectomy to sublobar resection, reducing size-threshold–defined potential overtreatment by 51.4% while maintaining sensitivity for true ≥ T2 tumors. Conclusions: CT demonstrates size-dependent discordance with pathological size; this discordance likely reflects both CT measurement inaccuracy and specimen shrinkage after fixation, and the relative contributions cannot be separated from these data. A candidate 23 mm CT threshold, supported by DCA and internal bootstrap validation, could reduce size-threshold–defined potential overtreatment by 51% in this cohort. Prospective multicenter validation is required before clinical implementation.

## Simple Summary

Surgeons rely on computed tomography (CT) scans to measure the size of lung tumors before deciding whether to remove an entire lobe of the lung (lobectomy) or a smaller portion (sublobar resection). The critical size boundary is 20 mm: tumors measured above this threshold are typically treated with the more extensive lobectomy. However, CT measurements do not always match the true tumor size found after surgery. In this study, we found that CT systematically overestimates the size of small tumors and underestimates large ones. As a result, approximately 16% of patients may have received a more extensive operation than their pathological tumor size would have indicated under a size-only decision rule. Using advanced statistical modeling, we identified 23 mm as a more appropriate CT threshold, which could reduce this excess surgery by over half while maintaining reliable detection of tumors that genuinely need lobectomy. These findings suggest that raising the CT decision boundary by 3 mm may reduce size-threshold–driven excess surgery in borderline cases, though prospective validation is needed before clinical implementation.

## 1. Introduction

Lung cancer remains the leading cause of cancer-related mortality worldwide, with non-small cell lung cancer (NSCLC) accounting for approximately 85% of all cases [1]. The widespread adoption of low-dose computed tomography (CT) has substantially increased the detection of early-stage lung cancer, particularly small peripheral nodules [2,3]. This shift toward earlier detection has transformed surgical treatment paradigms, with accumulating evidence supporting anatomical sublobar resection (segmentectomy or wedge resection) as a viable alternative to lobectomy for selected patients with small NSCLC [4,5,6].

Current clinical practice guidelines recommend lobectomy for tumors exceeding 20 mm in diameter, while anatomical sublobar resection may be considered for smaller lesions in patients with compromised pulmonary function or significant comorbidities [7,8]. However, the decision to perform either a lobectomy or an anatomical sublobar resection is dependent significantly on the preoperative CT measurements [9,10]. The critical assumption underlying this approach is that CT-measured tumor diameter accurately reflects pathological tumor size [11].

Despite the clinical significance of this issue, several key questions remain unanswered. The patterns of CT–pathology discordance across different tumor sizes have not been well defined in thoracoscopic surgery. The optimal CT cut-off for balancing sensitivity and specificity for surgical decision-making is undefined. Moreover, the real-world clinical consequences of overtreatment caused by CT overestimation have not been adequately quantified.

We hypothesized that CT-based tumor diameter systematically differs from pathological size near the 20 mm surgical boundary in a size-dependent manner, leading a significant proportion of patients to undergo more extensive resection than pathology would warrant, and that modestly raising the CT threshold could reduce this overtreatment without compromising identification of tumors that genuinely require lobectomy.

## 2. Materials and Methods

### 2.1. Study Design and Patients

This retrospective cohort study was conducted at a single tertiary care facility with the approval of the Institutional Review Board (IRB protocol number KY2025-103). The requirement for informed consent was waived by the Institutional Review Board due to the retrospective nature of the study and the use of de-identified clinical records. The study evaluated CT–pathology tumor size discordance and its clinical impact on surgical decision-making among patients undergoing robotic-assisted thoracoscopic surgery for primary lung cancer between January 2020 and December 2024.

Patients were included if they had a preoperative chest CT scan performed within 3 months prior to surgery and a complete pathological evaluation of lung cancer following thoracoscopic lobectomy or segmentectomy, with a pathological diagnosis of primary lung cancer. Surgical patients were included regardless of preoperative CT tumor size, provided that paired CT and pathological maximal tumor diameter measurements were available.

Patients were excluded if they had received neoadjuvant chemotherapy or radiation, had multiple primary lung cancers, had detectable distant disease at the time of thoracoscopic surgery, had incomplete medical records, or if their preoperative chest CT images were unavailable, of poor quality, or inadequate for accurate tumor diameter measurement. Of the 1185 patients initially enrolled, 89 (7.5%) were excluded due to missing CT measurements or imaging issues, including inaccessible images (n = 52, 58%), poor image quality (n = 24, 27%), or other technical problems (n = 13, 15%), resulting in a final cohort of 1096 patients.

### 2.2. CT Measurement and Clinical Staging

All patients underwent multi-detector CT scans of the thorax using thin-section imaging (slice thickness ≤ 2 mm) prior to surgery using standardized institutional lung cancer protocols (120 kVp, automated tube current modulation; scanner models: Siemens SOMATOM Definition Edge (Erlangen, Germany) and GE Revolution CT(GE HealthCare, Chicago, IL, USA)). CT image measurements were performed by two experienced thoracic radiologists (each with >10 years of experience) blinded to the pathological results. CT measurements were obtained using a mediastinal setting (window width: 350–450 HU; window level: 40–60 HU) at the slice showing the largest tumor diameter. For ground-glass opacity (GGO) lesions, both mediastinal and lung settings were utilized, with the maximum measurement reported. Discrepancies > 2 mm were resolved by consensus. Inter-reader agreement was excellent (ICC = 0.92; 95% CI, 0.89–0.94). The maximal total diameter was used for preoperative clinical staging per the 8th edition of the TNM classification system.

### 2.3. Surgical Procedure

All procedures were performed using the Da Vinci Robotic Surgical System (Intuitive Surgical, Sunnyvale, CA, USA). The choice of surgical approach was determined by the attending surgeon based on tumor size, location, patient cardiopulmonary reserve, and patient preferences. Patients with tumors > 20 mm or centrally located were generally treated with lobectomy; patients with clinically T1a–b tumors (≤20 mm) or T1c tumors (≤30 mm) with compromised lung function were treated with segmentectomy.

### 2.4. Pathological Evaluation

All surgical specimens were evaluated by experienced thoracic pathologists in accordance with the 8th edition of the TNM lung cancer classification system. Pathological tumor size was defined as the maximal total lesion diameter (total tumor size), measured on the resected specimen after routine fixation and sectioning. This definition was applied consistently to both lobectomy and segmentectomy specimens, including lesions with ground-glass components. Pathologists were blinded to the CT measurements.

### 2.5. Definition of Overtreatment

Patients were considered to have undergone size-threshold–discordant lobectomy (operationally termed “potential overtreatment”) if they underwent lobectomy despite having a maximal pathological total tumor diameter of ≤20 mm, under a size-only eligibility assumption. We acknowledge that lobectomy may still be clinically appropriate for some such tumors because operative strategy also depends on tumor location, multiplicity, anatomic constraints, margin feasibility, nodal assessment, and patient factors; these were not explicitly modeled in the present analysis.

For analyses evaluating CT decision thresholds, “CT-driven overtreatment” was further operationalized as lobectomy performed in patients with pathological total diameter ≤ 20 mm whose CT-measured diameter exceeded a given CT threshold T. “CT-driven undertreatment” was defined as sublobar resection in patients with pathological total diameter > 20 mm whose CT-measured diameter was ≤T. These terms are used strictly as decision-analytic labels relative to a size-only rule.

### 2.6. Statistical Analysis

Measurement bias was assessed using Bland–Altman methodology. The measurement difference was defined as Δ = CT diameter − pathological total diameter (mm), with positive values indicating CT overestimation. Mean bias, standard deviation, and 95% limits of agreement (LOA) were calculated per tumor size group (≤10 mm, 11–20 mm, 21–30 mm, >30 mm).

The optimal CT threshold was determined using a restricted cubic spline (RCS) regression model with four knots at the 5th, 35th, 65th, and 95th percentiles of the CT diameter distribution, with pathological diameter >20 mm as the binary outcome. Bootstrap resampling (B = 2000 replicates) was performed to validate threshold stability under both Youden index and net benefit maximization criteria.

Decision curve analysis (DCA) was conducted comparing CT > 20 mm versus CT > 23 mm versus treat-all and treat-none strategies. Net benefit was calculated as NB = (TP/n) − (FP/n) × [Pt/(1 − Pt)]. A 2 × 2 reclassification table was constructed comparing surgical decisions under the two thresholds. To evaluate CT–pathology agreement formally, paired Wilcoxon signed-rank tests were applied to compare CT and pathological diameters within each size stratum, with Hodges–Lehmann estimates and 95% confidence intervals reported. Spearman’s rank correlation coefficient (ρ) between CT and pathological diameter was calculated overall and by stratum. A sensitivity analysis applied a 1 mm measurement tolerance (clinically meaningful discordance defined as |CT − pathological diameter| > 1 mm). Internal validation of the RCS model was performed using bootstrap resampling (B = 200) via the rms package, yielding optimism-corrected C-statistics (apparent C = 0.881; optimism-corrected C = 0.880); model calibration was assessed with the Hosmer–Lemeshow test (χ^2^ = 13.35, df = 8, *p* = 0.100). A multivariable logistic regression model for lobectomy versus sublobar resection was fitted including CT diameter, age, sex, body mass index, FEV1, smoking history, and modified Charlson Comorbidity Index as covariates; model discrimination was assessed by the area under the receiver operating characteristic curve (AUC = 0.812). Statistical analyses were performed in R (version 4.3.2; R Foundation for Statistical Computing, Vienna, Austria) with the rms, dcurves, pROC, and ggplot2 packages. A two-tailed *p*-value < 0.05 was considered statistically significant.

## 3. Results

### 3.1. Baseline Characteristics

**Figure 1:**
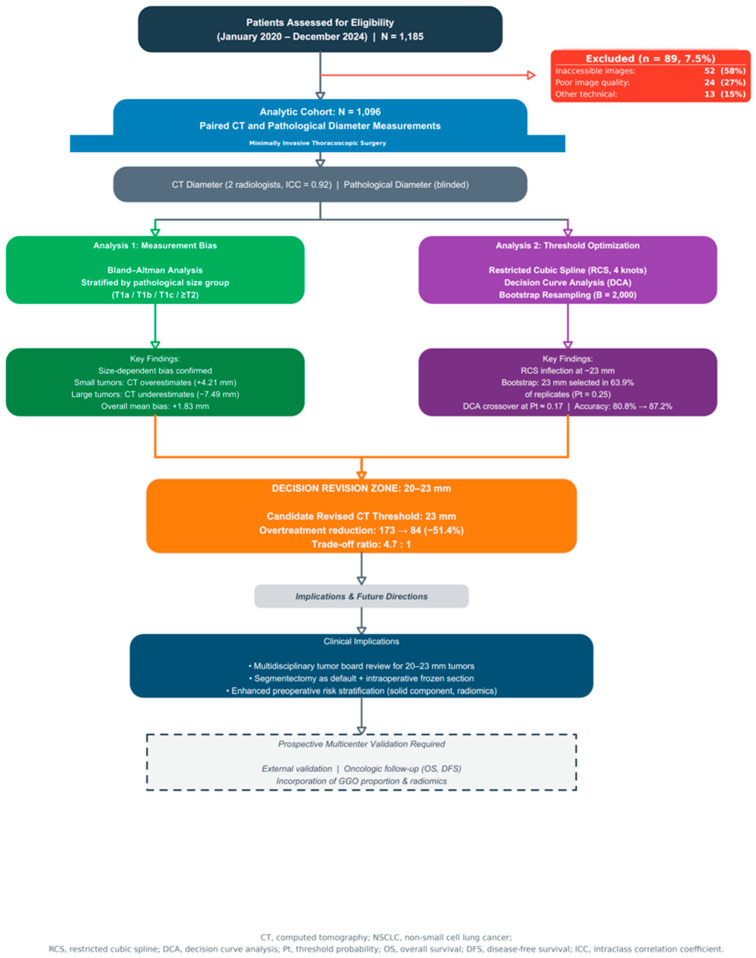
Study design and analysis framework. Of 1185 patients initially assessed, 89 (7.5%) were excluded due to unavailable or inadequate CT imaging, yielding an analytic cohort of 1096 patients with paired CT and pathological diameter measurements. Two parallel analyses were conducted: Analysis 1 evaluated CT–pathology measurement bias using Bland–Altman methodology. Analysis 2 performed threshold optimization using restricted cubic spline (RCS) modeling, decision curve analysis (DCA), and bootstrap resampling (B = 2000). Findings converged on a “decision revision zone” of 20–23 mm, with 23 mm identified as the candidate revised CT threshold. CT, computed tomography; RATS, robotic-assisted thoracoscopic surgery; NSCLC, non-small cell lung cancer; RCS, restricted cubic spline; DCA, decision curve analysis.

A total of 1185 patients underwent thoracoscopic surgery for NSCLC; 89 patients (7.5%) were excluded due to unavailable CT or pathological measurements. The final cohort comprised 1096 patients (mean age 59.5 ± 9.8 years; 38.1% male; mean BMI 24.06 ± 3.37 kg/m^2^). Smoking history was present in 190 patients (17.3%). Comorbidities included hypertension (n = 252, 23.0%), diabetes mellitus (n = 109, 9.9%), and coronary heart disease (n = 76, 6.9%). Lobectomy was performed in 574 patients (52.4%), sublobar resection in 522 (47.6%). Mean CT tumor diameter was 17.77 ± 10.04 mm, and mean pathological diameter was 15.39 ± 10.76 mm. Baseline characteristics are summarized in Table 1. The study design and analysis framework are illustrated in Figure 1.

### 3.2. Systematic Measurement Bias

**Figure 2:**
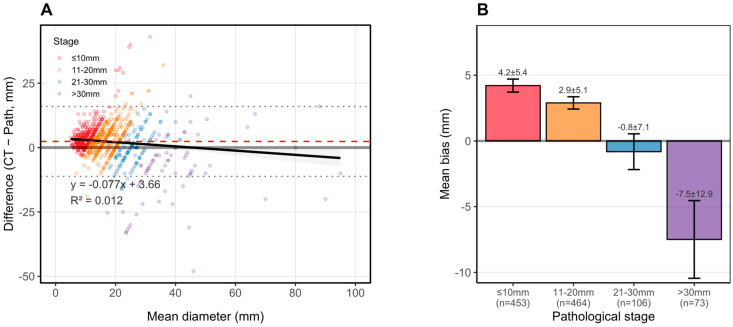
Systematic measurement bias between CT- and pathology-based tumor diameters. (**A**) Bland–Altman plot (linear fit: y = −0.077x + 3.66; R^2^ = 0.012). The solid line indicates mean bias; dashed lines indicate 95% limits of agreement. Points are colored by pathological stage. (**B**) Mean bias stratified by pathological tumor size group with 95% confidence intervals.

**Figure 3:**
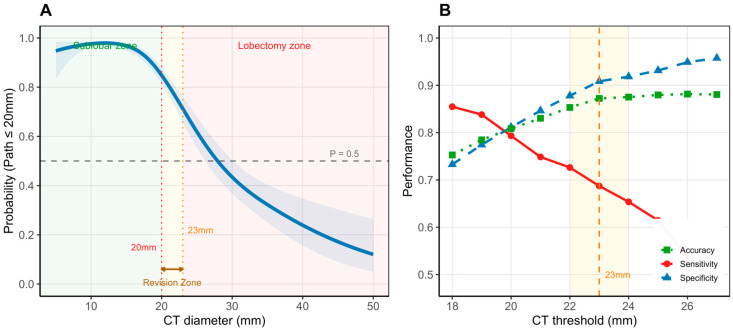
Threshold optimization and the decision revision zone. (**A**) Restricted cubic spline (RCS; 4 knots) depicting the nonlinear association between CT diameter and the probability of pathological diameter ≤ 20 mm. Shaded bands represent 95% confidence intervals. The double-headed arrow marks the 20–23 mm “decision revision zone.” (**B**) Diagnostic performance (accuracy, sensitivity, and specificity) across candidate CT thresholds, with a vertical line indicating the proposed 23 mm cutoff.

Bland–Altman analysis (Figure 2) revealed systematic size-dependent measurement bias. Overall mean bias was +1.83 mm. CT overestimated pathological size in smaller tumors and underestimated size in larger tumors: mean bias was +4.21 mm (SD 5.38; 95% LOA −6.33 to +14.75) for ≤10 mm tumors; +2.89 mm (SD 5.15; 95% LOA −7.20 to +12.98) for 11–20 mm tumors; −0.82 mm (SD 7.14) for 21–30 mm tumors; and −7.49 mm (SD 12.88) for >30 mm tumors. The proportion with CT overestimation decreased from 86.5% (≤10 mm) to 24.7% (>30 mm). Linear regression confirmed a negative association between mean tumor diameter and bias (Figure 3). Stratified Bland–Altman results are presented in Table 2.

### 3.3. Clinical Impact of the 20 mm Threshold

**Figure 4:**
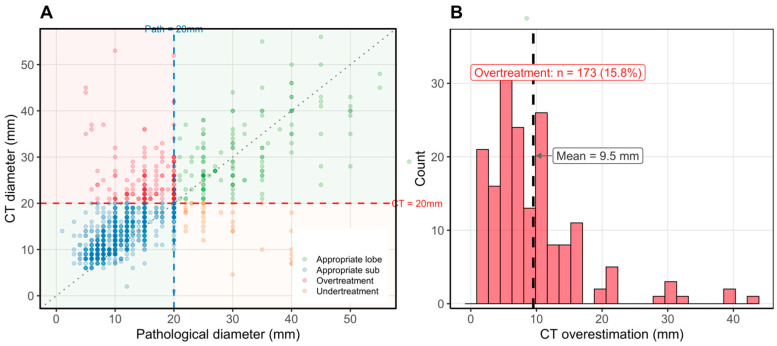
Clinical impact of misclassification at the 20 mm CT threshold. (**A**) Four-quadrant scatter plot of CT versus pathological maximal tumor diameter. Background shading denotes clinical impact zones (red: overtreatment; green: appropriate treatment; orange: undertreatment). (**B**) Histogram of CT overestimation among overtreatment cases (n = 173; mean overestimation 9.5 mm).

Using the traditional 20 mm cutoff, 173 out of 1096 patients (15.8%) were classified as potential overtreatment cases: patients with CT diameter > 20 mm but pathological diameter ≤ 20 mm who underwent lobectomy despite tumor size warranting sublobar resection under a size-only framework (Figure 4A). An additional 37 patients (3.4%) were classified as potential undertreatment (CT diameter ≤ 20 mm; pathological diameter > 20 mm). Mean CT overestimation in the overtreatment group was 9.5 ± 7.4 mm (Figure 4B). It should be noted that among these 173 patients, clinical or oncological factors beyond tumor size—such as central tumor location, anatomic constraints, or intraoperative nodal findings—may have independently justified lobectomy in a subset of cases; individual clinical audit was beyond the scope of this analysis.

### 3.4. Threshold Optimization and Decision Revision Zone

An RCS model (Figure 3A) illustrated a nonlinear relationship between CT diameter and the probability of pathological diameter ≤ 20 mm, with a clear inflection point at approximately 23 mm. This region (20–23 mm) was designated the “decision revision zone.” Raising the threshold from 20 mm to 23 mm improved overall diagnostic accuracy from 80.8% to 87.2% and reduced the number of overtreatment cases from 173 to 84 (a 51.4% reduction; Figure 3B). Specificity improved from 81.1% to 90.8%, while sensitivity remained acceptable at 68.7% (vs. 79.3% at 20 mm). Performance metrics at adjacent thresholds: 22 mm (sensitivity 72.6%, specificity 87.8%, accuracy 85.3%, overtreatment n = 112); 24 mm (sensitivity 65.4%, specificity 91.8%, accuracy 87.5%, overtreatment n = 75) (Table 3).

### 3.5. Bootstrap Validation of the Optimized Threshold

**Figure 5:**
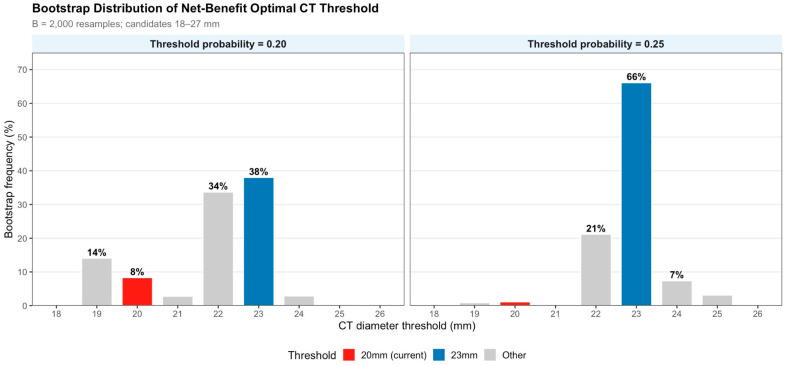
Bootstrap distribution of the net benefit optimal CT diameter threshold (B = 2000 resamples; candidate range 18–27 mm). Each bar represents the proportion of bootstrap replicates in which a given CT threshold maximized net benefit at the specified threshold probability. Left panel: threshold probability, Pt = 0.20; right panel: Pt = 0.25. Red bar: current 20 mm threshold; blue bar: proposed 23 mm threshold; grey bars: other candidates. At Pt = 0.25, 23 mm was selected in 63.9% of replicates (median 23 mm; 95% CI, 21–25 mm), demonstrating strong predominance under greater aversion to overtreatment.

Bootstrap resampling (B = 2000) evaluated threshold stability under Youden index and net benefit maximization criteria. Under the Youden criterion, the median optimal threshold was 20 mm (95% CI, 19–23 mm), with no single cutoff consistently dominant within the 19–23 mm interval. Under net benefit maximization at Pt = 0.20, the median threshold shifted to 22 mm (95% CI, 19–24 mm; 23 mm selected in 38.0% of replicates). At Pt = 0.25, the median was 23 mm (95% CI, 21–25 mm; selected in 63.9% of replicates), indicating strong predominance under greater aversion to overtreatment. Sensitivity analyses with expanded candidate ranges (10–40 mm) were consistent, supporting robustness (Table 4; Figure 5).

### 3.6. Decision Curve Analysis

**Figure 6:**
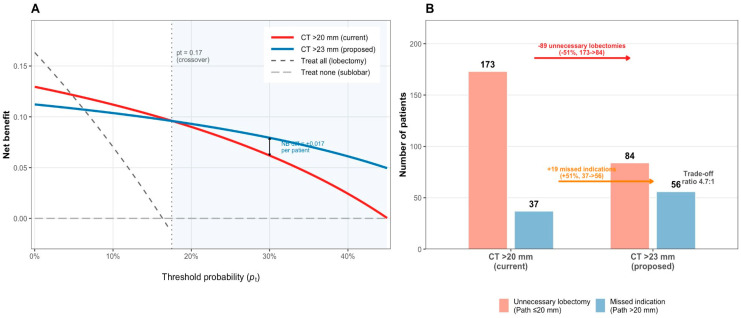
Decision curve analysis and reclassification impact of revising the CT threshold from 20 mm to 23 mm. (**A**) Decision curve analysis (DCA) comparing CT > 20 mm (current practice) versus CT > 23 mm (proposed) against treat-all and treat-none strategies. The curves cross at approximately Pt ≈ 0.17; at higher Pt values, CT > 23 mm yields greater net benefit. (**B**) Reclassification summary: raising the threshold reduced unnecessary lobectomies among patients with pathological diameter ≤ 20 mm from 173 to 84 (−89; −51.4%), while increasing missed lobectomy indications from 37 to 56 (+19). Trade-off ratio: 4.7:1.

DCA compared CT > 20 mm versus CT > 23 mm versus treat-all and treat-none strategies (Figure 6A). CT > 20 mm yielded slightly higher net benefit at Pt < 0.17, consistent with its higher sensitivity. At Pt ≥ 0.17, CT > 23 mm provided higher net benefit, with the advantage increasing with Pt (net benefit 0.093 vs. 0.090 at Pt = 0.20; 0.079 vs. 0.062 at Pt = 0.30). Both rules outperformed treat-all and treat-none reference strategies across the examined range. Net benefit values at key threshold probabilities are summarized in Table 5.

### 3.7. Reclassification Analysis

Under CT > 20 mm, 315 patients (28.7%) would receive lobectomy, under CT > 23 mm, 207 (18.9%). Adoption of 23 mm reclassified 108 patients from lobectomy to sublobar resection: 89 with pathological diameter ≤ 20 mm (unnecessary lobectomies avoided; 51.4% reduction in overtreatment from 173 to 84) and 19 with pathological diameter > 20 mm (missed indications; undertreatment increasing from 37 to 56). The trade-off ratio was 4.7:1—approximately 4.7 unnecessary lobectomies avoided per additional missed indication—consistent with the DCA crossover at Pt ≈ 0.17 (Figure 6B).

### 3.8. Diagnostic Performance

Table 6 reports diagnostic performance for CT > 20 mm and CT > 23 mm with bootstrap-derived 95% confidence intervals (B = 2000). Compared with CT > 20 mm, CT > 23 mm demonstrated higher specificity and positive predictive value (PPV) with a reduction in sensitivity. Negative predictive value (NPV) remained high for both rules (≥0.94), consistent with the low prevalence of pathological diameter >20 mm in this cohort (16.3%).

### 3.9. Multivariable Predictors of Surgical Approach

A multivariable logistic regression model predicting lobectomy versus sublobar resection was fitted to contextualize the role of CT diameter within a broader clinical decision framework (Table 7). CT diameter was the dominant predictor of surgical approach (OR per 1 mm: 1.21, 95% CI 1.17–1.24; *p* < 0.001), confirming that preoperative CT size is the primary driver of operative decisions in this cohort. Age (OR 1.02 per year; *p* = 0.033) and smoking history (OR 1.63 for ever vs. never; *p* = 0.018) were independently associated with lobectomy selection, while BMI showed a modest inverse association (OR 0.96 per kg/m^2^; *p* = 0.043). Sex, FEV1, and comorbidity burden (modified Charlson Comorbidity Index) were not independently predictive (all *p* > 0.05). The model demonstrated good discrimination (AUC = 0.812) and adequate calibration (Hosmer–Lemeshow: χ^2^ = 14.61, df = 8, *p* = 0.067). These findings confirm that CT diameter dominates operative decision-making and that the size-threshold–driven component of surgical assignment is the primary target of the proposed threshold revision.

## 4. Discussion

This study evaluated CT–pathology size discordance in a contemporary cohort of 1096 patients undergoing thoracoscopic surgery, demonstrating three clinically consequential findings. First, CT measurement bias is size-dependent: CT systematically overestimates smaller (≤20 mm) tumors and underestimates larger (>30 mm) lesions. Second, under a 20 mm size-only decision rule, 15.8% of patients were potential overtreatment cases, and 3.4% were potential undertreatment cases. Third, shifting the CT threshold to 23 mm reduces CT-driven overtreatment by 51.4% at a 4.7:1 trade-off, with superior decision-analytic utility at threshold probabilities ≥ 0.17.

The crossover from overestimation in T1a tumors (+4.21 mm) to underestimation in ≥T2 tumors (−7.49 mm) supports the “crossover” phenomenon reported in prior imaging series, quantified here in a contemporary thoracoscopic surgery cohort [12,13]. Overestimation of small lesions is consistent with prior reports attributing inaccuracies to volume averaging and difficulty delineating GGO–parenchyma interfaces [14,15]. Tissue processing contributes further: formalin fixation and post-resection deflation can induce 10–30% shrinkage [16,17], more pronounced in solid components than lepidic growth patterns [18,19,20].

A 15.8% CT-driven overtreatment rate is clinically consequential. The revision from 20 mm to 23 mm is best justified by decision-analytic utility rather than accuracy alone, since bootstrap results under Youden optimization do not identify a single consistently dominant cutpoint within 19–23 mm. A 3 mm adjustment is conceptually aligned with landmark trials (JCOG0802/WJOG4607L and CALGB 140503), which demonstrated non-inferiority of segmentectomy for tumors ≤ 20 mm [4,5]. Accumulating evidence also suggests oncologic adequacy of sublobar resection for selected T1c tumors with sufficient margins [21,22]. In our cohort, CT > 23 mm improved specificity and PPV while preserving strong rule-out performance (high NPV), primarily reducing false-positive “lobectomy indications”.

The selection of 23 mm rather than 22 mm or 24 mm as the upper boundary of the decision revision zone rests on three converging lines of evidence. First, bootstrap-based net benefit analysis at Pt = 0.25 identifies 23 mm as both the median and modal optimal cut-off (selected in 63.9% of replicates), whereas 22 mm is favored at Pt = 0.20 (median 22 mm; 23 mm modal at 38.0%); 23 mm thus represents the more robust choice across the plausible range of clinical decision weights. Second, 24 mm yields marginally higher specificity (91.8%) at the cost of sensitivity (65.4%), which may be clinically unacceptable in populations with higher prevalence of pathological size > 20 mm. Third, 23 mm aligns with the enrollment boundary of the landmark JCOG0802/WJOG4607L and CALGB 140503 trials (clinical T1a–b tumors, ≤20 mm), naturally defining the 20–23 mm CT range as a zone of clinical uncertainty not fully addressed by those trials [4,5]. We acknowledge that 22 mm remains a statistically defensible alternative; the decision revision zone (20–23 mm) is therefore best understood as a range warranting heightened clinical scrutiny rather than a single mandatory decision cut-off.

The apparent discrepancy between the Youden-optimal cut-off (median 20 mm; 95% CI, 19–23 mm) and the net benefit-optimal decision threshold (median 22–23 mm at Pt = 0.20–0.25) reflects a fundamental difference in optimization criteria rather than a methodological inconsistency. The Youden index treats false-positive and false-negative errors as equally costly; under this symmetric loss, the current 20 mm cut-off is near-optimal. However, in thoracic surgical decision-making, an unnecessary lobectomy carries substantially greater harm than a missed indication—due to increased perioperative morbidity, long-term reduction in pulmonary reserve, and absence of oncologic benefit for pathologically small tumors. Decision curve analysis incorporates this asymmetry explicitly through the threshold probability, Pt. At clinically plausible values of Pt ≥ 0.17, the CT > 23 mm rule yields higher net benefit, and bootstrap resampling confirms this advantage is robust (23 mm selected in 63.9% of replicates at Pt = 0.25). We therefore propose 23 mm not as the accuracy-optimal cut-off, but as the decision-analytic-optimal operating point under realistic clinical preferences in which overtreatment is weighted more heavily than undertreatment.

Clinically, unnecessary lobectomy for small tumors exposes patients to short-term risks without clear oncologic benefit [23,24]. Preserving lung parenchyma is increasingly important for postoperative quality of life and physiologic reserve, particularly in older patients at risk for metachronous lung cancer [25]. These considerations underscore why a modest upward revision of the CT threshold—applied specifically to borderline measurements—may improve the balance between benefit and harm in real-world surgical decision-making.

### Clinical Implications

These findings support a refined, precision-oriented surgical decision-making model applicable to contemporary minimally invasive thoracic surgery. For tumors measuring 20–23 mm on CT (the “decision revision zone”), several strategies may reduce CT-driven overtreatment: (1) Multidisciplinary tumor board discussion with explicit attention to CT overestimation in borderline-size tumors and interpretive nuances of GGO. (2) Anatomical segmentectomy as the default approach, paired with intraoperative frozen section to confirm margin adequacy and nodal status, with conversion to lobectomy reserved for inadequate margins or occult nodal involvement. (3) Enhanced preoperative risk stratification using solid-component measurement and radiomic signatures to improve staging reliability. Our DCA provides a practical anchor for shared decision-making. The threshold probability (Pt) represents the minimum probability of pathological size > 20 mm at which a clinician would recommend lobectomy. A Pt of 0.17 implies willingness to perform approximately one unnecessary lobectomy per five correctly identified large tumors; a Pt of 0.20 implies accepting up to four; a Pt of 0.25 implies no more than three. When Pt ≥ 0.17, CT > 23 mm yields higher net benefit; when Pt < 0.17, CT > 20 mm may be preferred. For patients who strongly value lung parenchyma preservation, a higher implicit Pt supports use of the 23 mm threshold.

## 5. Limitations

This study has several limitations. First, the retrospective, single-center design limits generalizability; external validation in multicenter cohorts is required, given inter-institutional variability in CT acquisition and reconstruction protocols [26]. Second, 7.5% of patients were excluded due to unavailable imaging data, potentially introducing selection bias. Third, CT–pathology discordance was not stratified by radiologic phenotype (pure GGO, part-solid, or solid nodules) or GGO proportion, nor were radiomics incorporated. Fourth, although inter-reader reliability was excellent (ICC = 0.92), automated AI-based segmentation tools were not evaluated. Fifth, operative strategy is not determined by size alone but also by tumor location, multiplicity, anatomic constraints, margin feasibility, and nodal assessment; because these determinants were not explicitly modeled, clinical translation may differ across practice settings. Sixth, long-term oncologic endpoints (overall survival and disease-free survival) were not assessed; the oncologic safety of shifting from 20 mm to 23 mm cannot be inferred from our data.

The aim of this study was not to revise existing guideline recommendations but to identify a statistically supported candidate CT threshold (23 mm) from real-world data to inform subsequent independent validation. We therefore suggest heightened caution when CT-measured diameter falls within the approximately 18–25 mm decision revision zone, where reliance on a single linear measurement may be suboptimal. In such borderline cases, multimodal imaging assessment, multidisciplinary review, and individualized patient and tumor factors should be incorporated into decision-making.

## 6. Conclusions

The observed CT–pathology discordance likely reflects both CT measurement inaccuracy and specimen shrinkage after fixation; the relative contributions cannot be disentangled from the present retrospective data. A candidate-revised CT cut-off of 23 mm—supported by RCS modeling, decision curve analysis, and internal bootstrap validation—may reduce CT-threshold-driven potential overtreatment near the 20 mm boundary and facilitate parenchyma-sparing surgical strategies. External multicenter validation, incorporation of key surgical decision variables, and prospective oncologic follow-up are needed before broad clinical implementation.

## Figures and Tables

**Table 1:** Baseline characteristics of study population (N = 1,096).

Characteristic	Value
Demographics	
Age (years), mean ± SD	59.5 ± 9.8
Male sex, n (%)	418 (38.1)
BMI (kg/m^2^), mean ± SD	24.06 ± 3.37
Smoking history, n (%)	190 (17.3)
Comorbidities	
Modified Charlson Comorbidity Index	0.14 ± 0.37
Hypertension, n (%)	252 (23.0)
Diabetes mellitus, n (%)	109 (9.9)
Coronary heart disease, n (%)	76 (6.9)
Pulmonary Function	
FEV1 (L), mean ± SD	2.44 ± 0.62
FEV1/FVC (%), mean ± SD	79.0 ± 8.2
MVV (L/min), mean ± SD	74.7 ± 24.8
DLCO (mmol/min/kPa), mean ± SD	6.19 ± 1.69
Tumor Characteristics	
CT diameter (mm), mean ± SD	17.77 ± 10.04
Pathological diameter (mm), mean ± SD	15.39 ± 10.76
Pathological Size Distribution	
≤10 mm (T1a), n (%)	453 (41.3)
11–20 mm (T1b), n (%)	464 (42.3)
21–30 mm (T1c), n (%)	106 (9.7)
>30 mm (≥T2), n (%)	73 (6.7)
Surgical Approach	
Lobectomy, n (%)	574 (52.4)
Sublobar resection, n (%)	522 (47.6)

BMI, body mass index; FEV1, forced expiratory volume in 1 s; FVC, forced vital capacity; MVV, maximum voluntary ventilation; DLCO, diffusing capacity of the lungs for carbon monoxide.

**Table 2:** Stratified Bland–Altman analysis of CT and pathological diameters (N = 1096).

Pathological Size	n	Mean Bias (mm)	SD (mm)	95% LOA (mm)	Overestimation (%)
≤10 mm (T1a)	453	+4.21	5.38	−6.33 to +14.75	86.5%
11–20 mm (T1b)	464	+2.89	5.15	−7.20 to +12.98	67.7%
21–30 mm (T1c)	106	−0.82	7.14	−14.82 to +13.18	40.6%
>30 mm (≥T2)	73	−7.49	12.88	−32.74 to +17.75	24.7%

Bias calculated as (CT diameter − Pathological diameter). LOA, limits of agreement; SD, standard deviation. Positive bias indicates CT overestimation.

**Table 3:** Diagnostic performance of CT thresholds for predicting pathological tumor ≤20 mm.

CT Threshold	Sensitivity	Specificity	Accuracy	Overtreatment (n)	Note
20 mm	0.793	0.811	0.808	173	Current Standard
22 mm	0.726	0.878	0.853	112	Balance Point
23 mm	0.687	0.908	0.872	84	Optimal Accuracy
24 mm	0.654	0.918	0.875	75	Conservative

Sensitivity: correctly identifying tumors > 20 mm pathologically. Specificity: correctly identifying tumors ≤ 20 mm pathologically. Overtreatment: patients who underwent lobectomy (CT > threshold) but had pathological diameter ≤ 20 mm.

**Table 4:** Bootstrap distribution of the optimal CT diameter threshold under Youden index and net benefit-based criteria (B = 2000 replicates).

Optimality Criterion	Threshold Probability (Pt)	Median optimal Threshold (mm)	95% CI (mm)	Most Frequent Value (Frequency)
Youden index	—	20	19–23	19 mm (45.0%)
Maximum net benefit	0.20	22	19–24	23 mm (38.0%)
Maximum net benefit	0.25	23	21–25	23 mm (63.9%)
Sensitivity analysis (candidates 10–40 mm)	0.20	22	19–24	23 mm (38.0%)
Sensitivity analysis (candidates 10–40 mm)	0.25	23	22–25	23 mm (66.1%)

CI, confidence interval; Pt, threshold probability. Main analysis: candidate range 18–27 mm. Sensitivity analysis: candidate range 10–40 mm.

**Table 5:** Net benefit of CT diameter threshold rules at key threshold probabilities (decision curve analysis, n = 1096).

Pt	CT > 20 mm Net Benefit	CT > 23 mm Net Benefit	Difference (23 − 20)	Net Reduction per 100 pts †	Favors
**0.05**	0.121	0.108	−0.013	−1.3	CT >20 mm
**0.10**	0.112	0.104	−0.008	−0.8	CT > 20 mm
**0.15**	0.102	0.099	−0.003	−0.3	CT > 20 mm
**0.20**	0.090	0.093	**+0.003**	+0.3	**CT > 23 mm**
**0.25**	0.077	0.087	**+0.010**	+1.0	**CT > 23 mm**
**0.30**	0.062	0.079	**+0.017**	+1.7	**CT > 23 mm**

† Net reduction in unnecessary lobectomies per 100 patients relative to CT > 20 mm, calculated as (NB 23 mm − NB 20 mm) × 100. NB, net benefit; Pt, threshold probability.

**Table 6:** Diagnostic performance of CT diameter thresholds for pathological diameter > 20 mm (bootstrap 95% CI, B = 2000).

Threshold	Sensitivity (95% CI)	Specificity (95% CI)	PPV (95% CI)	NPV (95% CI)
CT > 20 mm (current)	0.79 [0.74–0.85]	0.81 [0.79–0.84]	0.45 [0.40–0.50]	0.95 [0.94–0.97]
CT > 23 mm (proposed)	0.69 [0.62–0.75]	0.91 [0.89–0.93]	0.59 [0.53–0.66]	0.94 [0.92–0.95]

Bootstrap 95% CIs derived from 2000 resamples with replacement. PPV, positive predictive value; NPV, negative predictive value.

**Table 7:** Multivariable logistic regression model for lobectomy versus sublobar resection (n = 1096).

Variable	OR [95% CI]	*p* Value
**CT diameter (per 1 mm)**	**1.21 [1.17–1.24]**	**<0.001**
Age (per year)	1.02 [1.00–1.04]	0.033
Sex (Male vs. Female)	0.81 [0.56–1.17]	0.256
BMI (per kg/m^2^)	0.96 [0.92–1.00]	0.043
FEV1 (per L)	1.15 [0.85–1.56]	0.361
Smoking (Ever vs. Never)	1.63 [1.09–2.43]	0.018
Modified CCI (per unit)	0.83 [0.56–1.23]	0.349

Outcome: lobectomy (vs. sublobar resection). Reference categories: female (sex), never smoker (smoking). OR, odds ratio; CI, confidence interval; BMI, body mass index; FEV1, forced expiratory volume in 1 s; CCI, Charlson Comorbidity Index. Model AUC = 0.812; Hosmer–Lemeshow: χ^2^ = 14.61, df = 8, *p* = 0.067. Bold: CT diameter is the dominant predictor (*p* < 0.001).

## Data Availability

The data underlying this article are derived from routine clinical records held at the study institution and are not publicly available owing to patient confidentiality and institutional data governance policies. De-identified data may be made available upon reasonable request to the corresponding author, subject to approval by the Institutional Review Board and in compliance with applicable data protection regulations.
